# Degradation of Zearalenone by Essential Oils under *In vitro* Conditions

**DOI:** 10.3389/fmicb.2016.01224

**Published:** 2016-08-11

**Authors:** Adam Perczak, Krzysztof Juś, Katarzyna Marchwińska, Daniela Gwiazdowska, Agnieszka Waśkiewicz, Piotr Goliński

**Affiliations:** ^1^Department of Chemistry, Poznań University of Life SciencesPoznań, Poland; ^2^Department of Natural Science and Quality Assurance, Faculty of Commodity Science, Poznań University of Economics and BusinessPoznań, Poland

**Keywords:** essential oils, zearalenone, degradation, mycotoxin, HPLC analysis

## Abstract

Essential oils are volatile compounds, extracted from plants, which have a strong odor. These compounds are known for their antibacterial and antifungal properties. However, data concerning degradation of mycotoxins by these metabolites are very limited. The aim of the present study was to investigate the effect of essential oils (cedarwood, cinnamon leaf, cinnamon bark, white grapefruit, pink grapefruit, lemon, eucalyptus, palmarosa, mint, thymic, and rosemary) on zearalenone (ZEA) reduction under various *in vitro* conditions, including the influence of temperature, pH, incubation time and mycotoxin and essential oil concentrations. The degree of ZEA reduction was determined by HPLC method. It was found that the kind of essential oil influences the effectiveness of toxin level reduction, the highest being observed for lemon, grapefruit, eucalyptus and palmarosa oils, while lavender, thymic and rosemary oils did not degrade the toxin. In addition, the decrease in ZEA content was temperature, pH as well as toxin and essential oil concentration dependent. Generally, higher reduction was observed at higher temperature in a wide range of pH, with clear evidence that the degradation rate increased gradually with time. In some combinations (e.g., palmarosa oil at pH 6 and 4 or 20°C) a toxin degradation rate higher than 99% was observed. It was concluded that some of the tested essential oils may be effective in detoxification of ZEA. We suggested that essential oils should be recognized as an interesting and effective means of ZEA decontamination and/or detoxification.

## Introduction

*Fusarium* species and their toxic secondary metabolites – mycotoxins – are responsible for plant diseases and colonize cereal grains (including wheat, barley, rice, and maize) before harvest, causing significant yield losses and reducing their quality (Fletcher et al., 2006; Kalagatur et al., 2015). *Fusarium* mycotoxins currently considered of importance from the toxicological point of view include zearalenone (ZEA), trichothecenes (mainly deoxynivalenol) and fumonisins, and their occurrence is now regulated by EU legislations. Mycotoxins have a negative impact on the health of humans and animals such as hepatotoxic, haemotoxic, nephrotoxic, estrogenic, and genotoxic effects (Ghedira-Chekir et al., 1999; Riley et al., 2001; Gelderblom et al., 2002; Abid-Essefi et al., 2004). The toxicity of mycotoxins differs depending on the kind of toxin, dose ingested, exposure, gender and age of animals or humans (Przybylska-Gornowicz et al., 2015; Szabó-Fodor et al., 2015).

Zearalenone – a macrocyclic β-resorcyclic acid lactone – is known as a non-steroidal estrogen biosynthesized through a polyketide pathway by *Fusarium* spp., including *F. graminearum*, *F. culmorum*, *F. cerealis*, *F. equiseti*, and *F. semitectum* (Goliński et al., 2009). ZEA is detected in different cereals and – as a thermostable compound – is also present in some final grain products, such as breakfast cereals, bread, pasta, beer, or processed feeds (Bullerman and Bianchini, 2007). ZEA may cause physiological alterations of the reproductive tract of domestic animals, especially pigs (Zinedine et al., 2007; Hueza et al., 2014) and it is also connected with precocious puberty of prepubertal girls (Jakimiuk et al., 2009; Massart and Saggese, 2011). Apart from its estrogenic properties, haematotoxic and genotoxic effects are also reported (Abid-Essefi et al., 2004; Zinedine et al., 2007; Hueza et al., 2014).

The most effective method to control growth of fungi as well as mycotoxin biosynthesis is prevention including pre- and postharvest strategies. The main solution for controlling fungal diseases in crops is chemical fungicides, with several negative implications after their application, e.g., observed and induced resistance to introduced substances reducing pathogen growth (Steffens et al., 1996; Aguin et al., 2006; Ishii, 2006). Therefore there is a need to find alternative ways for the control of fungi on crops such as biological agents or natural substances including essential oils. There is also a tendency to use essential oils as preservatives in the food industry to protect food from pathogenic or spoilage microorganisms both alone and in combination with other preservative methods (Tiwari et al., 2009; Somolinos et al., 2010). This is due to their strong antimicrobial properties.

Essential oils – as natural products – are complex, volatile, fragrant substances commonly occurring in secretory tissue cells. These odorous oily liquids are plants’ aromatic secondary metabolites, mostly obtained by extraction or steam distillation of leaves, fruits, bark, flowers, buds, twigs, seeds, roots and other various plant organs (Bakkali et al., 2008). Essential oil in terms of composition is a mixture of 20–60 different chemical compounds such as ketones, aldehydes, esters, alcohols, terpenes, terpenoids, lactones, and other organic substances. The amount as well as the presence of various ingredients in the essential oil depends mainly on the type of its source material, variety and quality. Major components determine properties of each essential oil, as these natural substances show a broad spectrum of antagonistic activity (Bakkali et al., 2008; Nerio et al., 2010).

*In vitro* and *in vivo* assays have proved that essential oils exhibit broad spectrum of application including cosmetic (Arung et al., 2011; Bertuzzi et al., 2013; Binic et al., 2013), antibacterial (Prabuseenivasan et al., 2006; Soković et al., 2010; Elaissi et al., 2012), fungicidal (Velluti et al., 2004), antiparasitical (Moon et al., 2006), antiprotozoal (Escobar et al., 2010), medical (Warnke et al., 2009; Buckle, 2014), insecticidal, sanitary (Ayvaz et al., 2010), repellent (Nerio et al., 2010) and agricultural (Lo Cantore et al., 2009) as well as in the food industry (Burt, 2004; Bakkali et al., 2008; Prakash et al., 2015). Several studies have reported the significant antifungal as well as antimycotoxigenic effect of essential oils (Tyagi and Malik, 2010; Xing et al., 2014; Rao et al., 2015). The above compounds are also considered as natural alternative to preservatives (Burt, 2004; Speranza and Corbo, 2010; Prakash et al., 2015).

Recently application of essential oils in prevention against fungi and mycotoxin production was reported (Aldred et al., 2008). Volatile essential oil of *Salvia fruticosa* demonstrated an antifungal effect against *Rhizoctonia solani, Sclerotinia sclerotiorum* and *Fusarium solani* (Pitarokili et al., 2003). On the other hand essential oils of *Zataria multiflora, Cuminum cyminum, Foeniculum vulgare, Pinaceae* and *Heracleum persicum* had an inhibitory effect on 11 non-toxigenic (*F. solani* and *F. oxysporum*) and 10 toxigenic (*F. verticillioides, F. poae* and *F. equiseti*) isolates (Naeini et al., 2010). In contrast, Velluti et al. (2003) found that cinnamon, clove, oregano, lemongrass and palmarosa essential oils inhibited growth of *F. proliferatum* and had an inhibitory effect on fumonisin B_1_ biosynthesis. The above were also recognized as inhibiting the growth rate of *F. graminearum*, ZEA and DON synthesis, although their inhibitory effect on the toxin production depended on environmental conditions (Velluti et al., 2004). It is also worth stressing that inhibition of fungal growth and mycotoxin biosynthesis does not always take place at the same time. Hope et al. (2005) found that cinnamon oil was effective in controlling growth of *F. culmorum* and *F. graminearum*, but enhanced toxin formation, which confirms that mycotoxin biosynthesis is associated with the primary metabolism pathway (energy).

Recently, some reports have indicated the possibility of mycotoxin degradation by essential oils, e.g., reduction of fumonisin B_1_ by cinnamon oil (Xing et al., 2014) or ochratoxin A by (among others) eucalyptus or neem oils (Rao et al., 2015). However, the data concerning detoxification of mycotoxins with these odorous natural complex compounds are limited, insufficient and need to be elucidated. Therefore the aim of the study was to evaluate the influence of different essential oils on degradation of ZEA under *in vitro* conditions.

## Materials and Methods

### Chemicals

Zearalenone standard was purchased with a standard grade certificate from Sigma–Aldrich (Steinheim, Germany). Organic solvents (HPLC grade) and all the other chemicals were also purchased from Sigma–Aldrich (Steinheim, Germany). Water for the HPLC mobile phase preparation was purified using a Milli-Q system (Millipore, Bedford, MA, USA). The stock solution of ZEA was prepared in methanol (1 mg/mL) and stored at -20°C. In experiment, three buffer solutions were used and prepared on the basis of citrate (pH 3 and 6) and ammonia (pH 9) buffer solutions.

### Essential Oils

Eleven essential oils were used in the study: cedarwood (*Juniperus virginiana*, USA), cinnamon leaf oil (*Cinnamomum zeylanicum*, Sri Lanka), cinnamon bark (*Cinnamomum zeylanicum*, Indonesia), lemon peel (*Citrus limonum*, Italy), pink grapefruit peel (*Citrus paradisi*, Argentina), white grapefruit peel (*Citrus grandis*, Argentina), lavender flower (*Lavandula angustifolia*, France), eucalyptus leaf oil (*Eucalyptus radiata*, China), thyme flower and leaf (*Thymus vulgaris*, Spain), rosemary flower and leaf (*Rosmarinus officinalis*, Spain) and palmarosa leaf oil (*Cymbopogon martinii*) (Ecospa Rita Kozak-Chaber Artur Chaber s.c., Poland). Solutions of essential oils were prepared by mixing with water and Tween 80 (10%) as an emulsifying agent. Depending on the experiment, a concentration of 100 and 200 μl/mL of essential oils was used.

### Effect of Essential Oils on Zearalenone Degradation

The effect of eleven different essential oils was examined by mixing ZEA with solutions of oils (100 μl/mL) in a concentration of 5 μg/mL. The mixture contained 100 μl of essential oil, 100 μl of Tween 80, 5 μl of ZEA stock solution and buffer solution at pH = 6 to a final volume of 1 mL. Samples were shaken and incubated for 72 h at 20°C and the concentration of toxin was assayed at 0, 24, and 72 h by HPLC analysis.

### Effect of Temperature, pH, Concentration of Essential Oils and/or Zearalenone on the Toxin Degradation

For the evaluation of the effect of different factors on ZEA degradation, seven essential oils were chosen: cinnamon leaf, cinnamon bark, lemon peel, pink grapefruit peel, white grapefruit peel, eucalyptus leaf and palmarosa leaf. Different process conditions temperature of samples’ incubation (4 and 20°C), pH (3, 6, and 9), concentration of essential oils (100 and 200 μl/mL) and concentration of the toxin (0.5 and 5 μl/mL of ZEA) were investigated while the time of incubation was 72 h. The mixture contained 100 μl (or 200 μl) of essential oil, 100 μl of Tween 80, 0.5, or 5 μl of ZEA stock solution and appropriate buffer solution to final volume 1 mL.

### HPLC Analysis

After incubation time, 1 mL of each reaction mixture was homogenized for 3 min with 5 mL of acetonitrile:water (90:10, v/v). ZEA was extracted and purified on a ZearalaTest column (Vicam, Milford, CT, USA) according to a procedure described in detail previously (Goliński et al., 2010). The elute was evaporated to dryness at 40°C under a stream of nitrogen. Dry residue was stored at -20°C until HPLC analyses. Evaporated extracts were dissolved in a 200 μL mixture of acetonitrile:methanol:water (70:20:10, v/v/v), homogenized in an ultrasonic bath (Ultron, type U-505, Dywity, Poland), filtered through a syringe filter of 0.2 μm mesh and applied onto the chromatographic column.

The chromatographic system used in the study consisted of a Waters 2695 high-performance liquid chromatograph (Waters, Milford, CT, USA) with detectors – Waters 2475 Multi aaa Fluorescence Detector (aaaex = 274 nm, aaaem = 440 nm) and Waters 2996 Photodiode Array Detector – and a Nova Pak C-18 column (150 × 3.9 mm). Data were processed using the Empower software (Waters, Milford, CT, USA). Quantification of ZEA was performed by measuring the peak areas at the retention time according to the relevant calibration curve. A Photodiode Array Detector (PDA) was used to confirm the presence of ZEA on the basis characteristic spectra of this compound. The limit of detection was 0.01 μg m L^-1^.

### Statistical Analysis

The presented data are the mean (±standard deviation) of three replicate trials and obtained results of the mycotoxin degradation were subjected to Student’s *t*-test at *p* < 0.05 to test for significant differences between different tested samples. The influence of above-mentioned process conditions (pH, temperature, essential oil concentrations) on ZEA degradation was examined by multivariate analysis of variance (ANOVA). Analyses were carried out using STATISTICA for Windows version 10.

## Results

### Effect of Essential Oils (EO) on Zearalenone Degradation Depending on Incubation Time

**FIGURE 1:**
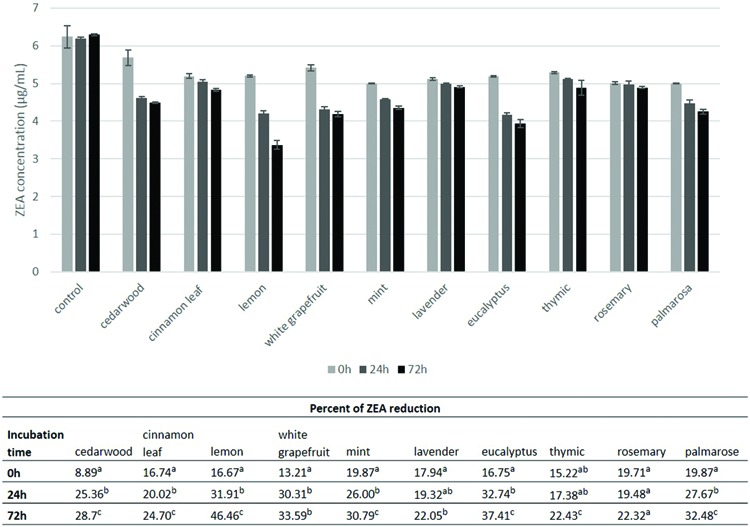
**Reduction of zearalenone content by essential oils at different incubation time.** The experiment was conducted up to 72 h at 20°C. Amount of zearalenone was determined by HPLC method. The concentration of essential oils was 100 μl/mL. Data were analyzed by Student’s *t*-test at *p* < 0.05 (a, b, c – significantly different in column).

In the first part of our work ten essential oils were tested for their ability to reduce ZEA content (**Figure 1**). All oils except lavender, thymic and rosemary oils reduced the concentration of toxin. Lemon, grapefruit, eucalyptus, and palmarosa oils were the most effective and the most interesting results were observed for lemon oil, as the amount of the toxin was reduced by up to 31.93% and 46.46% after 24 and 72 h, respectively. Cinnamon leaf essential oil only slightly, but statistically significantly, reduced ZEA by 20.02% and 24.70% after 24 and 72 h, respectively. Incubation time had a significant positive effect on ZEA degradation by tested essential oils. The greatest reduction of ZEA content was observed after 24 h of incubation. During the next 48 h toxin degradation was still observed, but the degree of reduction was lower. For example, cedarwood, grapefruit and eucalyptus essential oils reduced the amount of toxin by 25.36, 30.30, and 32.74% after 24 h of incubation, respectively, while after the next 48 h reduction by several percent was observed, reaching values of 28.73, 33.59, and 37.41%.

### Effect of EO Concentration on ZEA Degradation

**FIGURE 2:**
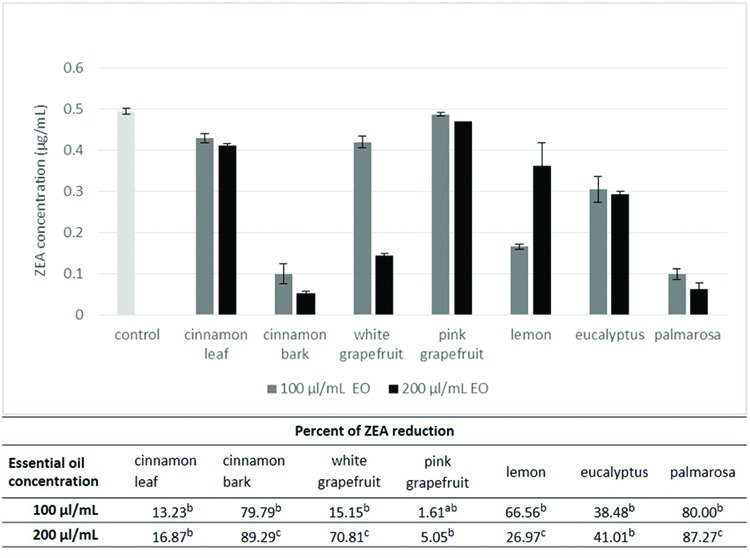
**The effect of the essential oils concentration on degradation of zearalenone.** The experiment was conducted for 72 h at 20°C. Amount of zearalenone was determined by HPLC method. The concentration of EO was 100 and 200 μl/mL. Data were analyzed by Student’s *t*-test at *p* < 0.05 (a, b, c – significantly different in column as well as in relation to control trial).

On the basis of first stage results, five essential oils were chosen for the next step: cinnamon, grapefruit, lemon, eucalyptus and palmarosa. Preliminary results did not indicate high effectiveness in reduction of ZEA content by cinnamon oil, but it was chosen mainly due to current literature data. Taking into account the differences between essential oils according to their origin, the study was expanded to include additional variants in the case of grapefruit and cinnamon oil. In the experiment the ZEA concentration used was 0.5 μg/mL assuming that a lower amount of toxin would reveal greater differences concerning the influence of different concentrations of essential oils. The results showed that the concentration of essential oil significantly influenced degradation of ZEA by cinnamon bark, white grapefruit, lemon and palmarosa oil (**Figure 2**). The greater the amount of oil, the higher was the rate of reduction of toxin concentration. However it is worth underlining that lemon oil was more efficient at its 10% addition to the samples. Nevertheless, the exact mechanism of such behavior is difficult to explain. Three of the tested essential oils, cinnamon leaf, pink grapefruit and eucalyptus, reduced ZEA with a similar rate, independently of the content of oil in the sample.

### Effect of pH, Temperature and Zearalenone Concentration on Toxin Degradation

**FIGURE 3:**
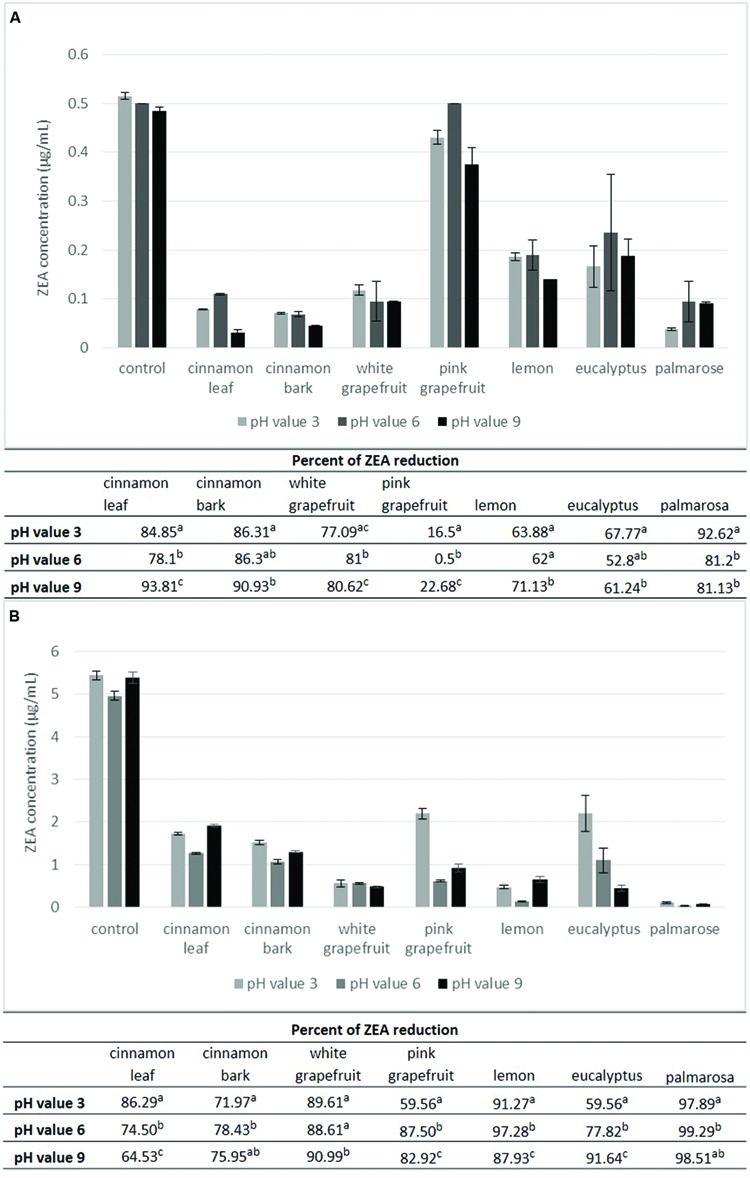
**The effect of the pH and zearalenone concentration on the toxin degradation at temperature 20°C.** The experiment was conducted 72 h at the toxin concentration level 0.5 μg/mL **(A)** and 5 μg/mL **(B)** and 100 μl/mL of essential oil. Amount of zearalenone was determined by HPLC method. Data were analyzed by Student’s *t*-test at *p* < 0.05 (a, b, c – significantly different in column).

**FIGURE 4:**
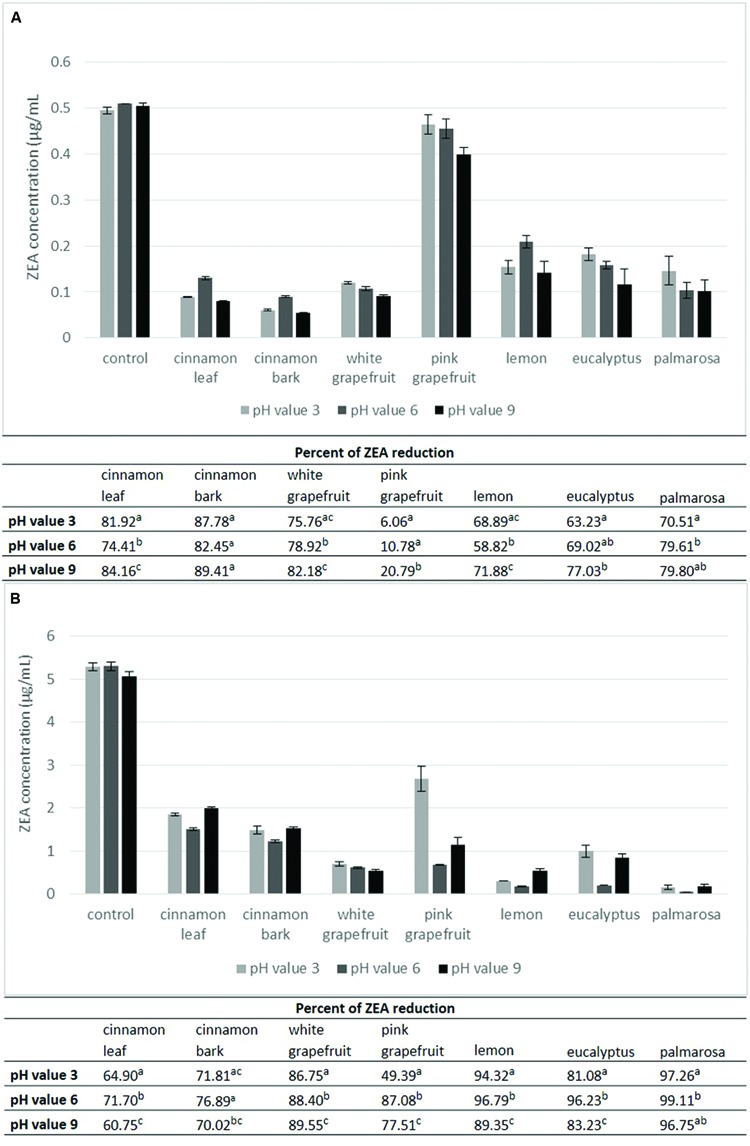
**The effect of the pH and zearalenone concentration on the toxin degradation at temperature 4°C.** The experiment was conducted 72 h at the toxin concentration level 0.5 μg/mL **(A)** and 5 μg/mL **(B)** and 100 μl/mL of essential oil. Amount of zearalenone was determined by HPLC method. Data were analyzed by Student’s *t*-test at *p* < 0.05 (a, b, c – significantly different in column).

Zearalenone degradation by selected essential oils under different conditions including pH (3, 6 and 9), temperature (4 and 20°C) and ZEA concentration (0.5 and 5 μg/mL) was investigated (**Figures 3** and **4**). All examined parameters influenced the effectiveness of ZEA degradation, but it depended on the kind of EO. The results presented in **Figure 3** show that the degree of ZEA reduction at 20°C was generally higher in samples containing 5 μg/mL (**Figure 3B**) and ranged from 59.56 to 99.29%, depending on the pH value as well as the EO used. At a concentration of 0.5 μg/mL (**Figure 3A**) the percentage of ZEA reduction showed greater variation. The weakest effect was observed for the pink grapefruit EO, where the highest percentage reduction was 22.68% at pH 9. The remaining essential oils reduced the amount of toxin by 52.8–93.81%. The value of pH at 20°C significantly influenced the ZEA degradation by all essential oils at a concentration of 5 μg/mL, but at a concentration of 0.5 μg/mL the effect of pH was not observed for palmarosa and eucalyptus oils. Considering the degradation degree of ZEA depending on the pH value it was noted that the highest reduction of toxin amount at the initial concentration of 0.5 μg/mL (**Figure 3A**) was observed at pH 9, excluding eucalyptus and palmarosa oils, which were more effective at pH 3. The lowest degradation rate was observed at pH 6 for all essential oils except for white grapefruit, which was the least effective at pH 3. At a concentration of 5 μg/mL (**Figure 4B**) such a dependency was not observed. Cinnamon bark, pink grapefruit, lemon and palmarosa oils demonstrated the highest reduction of ZEA amount at pH 6, white grapefruit and eucalyptus oils at pH 9 and cinnamon leaf oil at pH 3.

**Figure 4** presents results concerning the influence of the essential oils on the degradation rate of ZEA at 4°C depending on the pH value and toxin concentration. At a concentration of 0.5 μg/mL (**Figure 4A**) the percentage of toxin reduction ranged from 6.06 to 89.41%, while at 5 μg/mL (**Figure 4B**) the degradation rate ranged from 49.39 to 99.11%, depending on the kind of EO and pH value. Similarly to the effect observed at 20°C, it was found that the pH value at 4°C significantly influenced the ZEA degradation by all essential oils at both concentrations, excluding palmarosa and eucalyptus oils at a concentration of 0.5 μg/mL. Taking into account the degree of ZEA reduction depending on the pH value, it was noted that the highest degradation rate at the initial concentration of 0.5 μg/mL (**Figure 4A**) was observed at pH 9 for all essential oils. The smallest decrease of toxin amount showed greater variation. Four essential oils – white grapefruit, pink grapefruit, eucalyptus and palmarosa were least effective at pH 3, while three of them – cinnamon leaf, cinnamon bark and lemon oils – were least effective at pH 6. At the initial concentration of 5 μg/mL (**Figure 4B**) the influence of essential oils on the degradation rate of ZEA was more variable than that observed at 20°C. The highest effectiveness in reducing the toxin amount was observed at pH 6 for all essential oils except for white grapefruit, which was most effective at pH 9. The weakest effect of essential oils on ZEA degradation was observed at pH 9 in the case of cinnamon oils (leaf and bark) as well as lemon and palmarosa oils, while grapefruit oils (pink and white) and eucalyptus oil were least effective at pH 3.

Considering the effect of temperature on the level of ZEA degradation, it is worth underlining that the highest percentage reduction was observed at 20°C. In the majority of samples the toxin degradation effectiveness was higher by a few percent at 20°C, when compared to 4°C.

Considering the mycotoxin concentration and pH value, ZEA degradation abilities of essential oils may be ranked in decreasing order as shown in **Table 1**. The highest degradation rate in samples at ZEA concentration of 0.5 μg/mL was achieved for cinnamon bark oil (82.45–90.93%), while in samples with 5 μg/mL of ZEA palmarosa oil caused the highest metabolite reduction (96.75–99.29%).

**Table 1:** Degradation properties of essential oils (100 μl/mL) depending on the temperature, pH and toxin concentration.

Temp.	pH	Degradation properties of essential oils in decreasing order
**ZEA concentration = 0.5 μg/mL**		

20	3	**palmarosa** > cinnamon bark > cinnamon leaf > white grapefruit > eucalyptus > lemon > pink grapefruit
	6	cinnamon bark > white grapefruit and **palmarosa** > cinnamon leaf > lemon > eucalyptus > pink grapefruit
	9	cinnamon leaf > cinnamon bark > **palmarosa** > white grapefruit > lemon > eucalyptus > pink grapefruit
4	3	cinnamon bark > cinnamon leaf > white grapefruit > **palmarosa** > lemon > eucalyptus > pink grapefruit
	6	cinnamon bark > **palmarosa** > white grapefruit > cinnamon leaf > eucalyptus > lemon > pink grapefruit
	9	cinnamon bark > cinnamon leaf > white grapefruit > **palmarosa** > eucalyptus > lemon > pink grapefruit

**ZEA concentration = 5 μg/mL**		

20	3	**palmarosa** > lemon > white grapefruit > cinnamon bark > cinnamon leaf > pink grapefruit and eucalyptus
	6	**palmarosa** > lemon > white grapefruit > pink grapefruit > cinnamon bark > eucalyptus > cinnamon leaf
	9	**palmarosa** > eucalyptus > white grapefruit > lemon > pink grapefruit > cinnamon bark > cinnamon leaf
4	3	**palmarosa** > lemon > white grapefruit > eucalyptus > cinnamon bark > cinnamon leaf > pink grapefruit
	6	**palmarosa** > lemon > eucalyptus > white grapefruit > pink grapefruit > cinnamon bark > cinnamon leaf
	9	**palmarosa** > white grapefruit > lemon > eucalyptus > pink grapefruit > cinnamon bark > cinnamon leaf

## Discussion

Mycotoxins generate several problems because due to their physico-chemical stability the metabolites are transported and introduced into successive steps of the food chain. Therefore elimination of mycotoxins is of prime concern in research and food safety programs. Methods of detoxification of the above toxic metabolites include physical, chemical and biological treatment. One effective methods for ZEA detoxification is microbial degradation by species of *Rhodococcus, Clonostachys, Trichosporon* or *Nocardia* (El-Sharkawy and Abul-Hajj, 1988a; Duvick and Rood, 1998; Molnar et al., 2004). Microorganisms may transform toxins to metabolites of lower estrogenic properties, e.g., *Cunninghamella bainieri* converts ZEA to 2,4-dimethoxyl ZEA (El-Sharkawy and Abul-Hajj, 1988b) and *Rhizopus arrhizus* transforms ZEA to ZEA 4-sulfate (El-Sharkawy et al., 1991). However, it is worth underlining that biotransformation of ZEA may metabolize the molecule to compound(s) of comparable or even higher estrogenic effects. For example, *Rhizopus* sp. and *Alternaria alternata*, chemically transform ZEA to *α*- and β-zearalenol, without a decrease in oestrogenicity (Kamimura, 1986; El-Sharkawy et al., 1991), while El-Sharkawy and Abul-Hajj (1988b) reported conversion of ZEA by *Aspergillus ochraceous* to *α*- and β-zearalanols with estrogenic properties at a higher level when compared to ZEA. There are limited data concerning the possibilities of mycotoxin degradation by essential oils. In our studies 8 of 11 tested essential oils reduced the content of ZEA in the model experiment, with the highest effectiveness demonstrated by lemon, grapefruit, eucalyptus and palmarosa oils. Lavender, thymic and rosemary oils did not degrade the examined toxin. Similarly, Xing et al. (2014) reported that six of seven oils degraded fumonisin B_1_ and the most effective was cinnamon oil, followed by citral, eugenol, eucalyptus and camphor oil, while peppermint oil was ineffective in their studies.

We have observed that effectiveness of ZEA degradation depends on several factors including incubation time, temperature, pH and both essential oil and toxin concentrations. Temperature and pH had a significant effect on ZEA (at ZEA concentration of 5 μg/mL) degradation by all tested essential oils except palmarosa. In the case of lower ZEA content the influence of incubations conditions was more complex. Xing et al. (2014) reported that incubation time as well as temperature and concentration of cinnamon oil influenced the degradation of fumonisin B_1_. The authors stated that the degradation rate of toxin increased gradually with the increasing temperature in the range of 20–35°C. The degradation rate of fumonisin B_1_ was incubation time dependent and increased gradually with incubation time (up to 120 h). In our studies, a higher degradation rate was also observed at a higher temperature. Moreover, we also found that degradation effectiveness increased with incubation time (up to 72 h). The essential oil concentration effect, in contrast was dependent on the kind of the oil. This parameter was significant for cinnamon bark, white grapefruit, lemon and palmarosa oil. Similar observations were reported for the cinnamon oil concentration effect in the research of Xing et al. (2014). Their results showed that the content of the essential oil was higher the greater was the toxin degradation rate. Finally, the authors found that the optimal conditions for reduction of fumonisin B_1_ were temperature 30°C, incubation time 120 h and concentration of essential oil 280 μg/mL. The final toxin content was decreased by 94.06% (from 15.03 to 0.89 μg/mL).

In our research there were also observed significant differences in the degradation rate between tested essential oils. This could be due to different composition of essential oils and possible relationships (e.g., synergistic or antagonistic effects) between the components. Essential oils are very complex mixtures of different constituents and may contain 20 – 60 compounds. Their antimicrobial and antioxidant properties are usually associated with substances such as α-pinene, β-pinene, γ-terpinene and *p*-cymene, carvacrol, eugenol, geraniol, thymol, vanillic acid, camphor, linalool *trans*-cinnamaldehyde and *trans*-cinnamic acid (Marino et al., 2001; Delaquis et al., 2002; Si et al., 2006; Sonboli et al., 2006; Goze et al., 2009). Generally, components are included in two main groups with one formed on the basis of terpene and terpenoid chemical structure and the second formed on the basis of aromatic and aliphatic compounds (Bakkali et al., 2008). Authors often describe the relationship between structure of compounds dominating in the essential oils and their antifungal activity. For example, Bluma et al. (2008) reported that antifungal activity of tested oils was associated with monoterpenic phenols, mainly thymol eugenol and carvacrol. Moreover, it has been reported that antifungal properties are not related to one specific component but are associated rather with a mixture of constituents included in the essential oil (Ranasinghe et al., 2002; Hashem et al., 2010). Considering that there is a lack of data concerning the mode of action of essential oils as degrading factors and participation of individual components, the investigation presented here is planned to be continued.

## Conclusion

To our best knowledge this is the first report on degradation of ZEA by essential oils. Our results reveal that essential oils may reduce the content of toxin under a wide spectrum of process conditions. The results presented here as well as the literature data indicate that antifungal as well as antimycotoxigenic activity of essential oils should not be generalized based on these trials. It is difficult to indicate which essential oil has the highest degradation properties due to the variety of factors influencing the effectiveness of the process. However, based on the obtained results it can be stated that palmarosa EO showed good properties in reducing ZEA content. In contrast, in most cases the lowest ZEA degradation properties were demonstrated by pink grapefruit oil.

To obtain a general picture of essential oils’ mycotoxin degradation potential and abilities, larger scale laboratory studies must be conducted involving a large range of mycotoxins as well as detailed information on quantitative and qualitative composition and activity of individual components of oils. It would be useful to expand the range of essential oils including their various composition, varying their number and ratio, to draw more precise conclusions more useful in practice. Complex studies are needed to fully understand the mechanism of action of essential oils as well as the mode of their application considering protection of foods and feeds.

Essential oils seems to be a good alternative to other detoxification method since they are recommended as safe substances by the Food and Drug Administration (FDA, 2015). Moreover, these volatile compounds are recognized as part of the concept of “green pesticides,” which favors the use of environmentally friendly, natural substances rather than synthetic chemical compounds.

## Author Contributions

Design of the work: DG, AW, KJ, AP, KM, and PG. Conducting experiments: AP, KJ, and KM. Interpretation of data: AP, KJ, KM, DG, AW, and PG. Drafting the work: AP, KJ, KM, DG, AW, and PG. Final approval: DG, AW, and PG.

## Conflict of Interest Statement

The authors declare that the research was conducted in the absence of any commercial or financial relationships that could be construed as a potential conflict of interest.
